# Communications Technology and Motor Neuron Disease: An Australian Survey of People With Motor Neuron Disease

**DOI:** 10.2196/rehab.4017

**Published:** 2016-01-25

**Authors:** Lynette Mackenzie, Prarthna Bhuta, Kim Rusten, Janet Devine, Anna Love, Penny Waterson

**Affiliations:** ^1^Faculty of Health SciencesDiscipline of Occupational TherapyUniversity of SydneyLidcombeAustralia; ^2^Motor Neurone Disease Association of NSW (MND NSW)GladesvilleAustralia

**Keywords:** Internet, tablet, referral and consultation, telemedicine, electronic mail, social support, amyotrophic lateral sclerosis, assistive technology, augmentative and alternative communication

## Abstract

**Background:**

People with Motor Neuron Disease (MND), of which amyotrophic lateral sclerosis (ALS) is the most common form in adults, typically experience difficulties with communication and disabilities associated with movement. Assistive technology is essential to facilitate everyday activities, promote social support and enhance quality of life.

**Objective:**

This study aimed to explore the types of mainstream and commonly available communication technology used by people with MND including software and hardware, to identify the levels of confidence and skill that people with MND reported in using technology, to determine perceived barriers to the use of technology for communication, and to investigate the willingness of people with MND to adopt alternative modes of communication.

**Methods:**

An on-line survey was distributed to members of the New South Wales Motor Neuron Disease Association (MND NSW). Descriptive techniques were used to summarize frequencies of responses and cross tabulate data. Free-text responses to survey items and verbal comments from participants who chose to undertake the survey by telephone were analyzed using thematic analysis.

**Results:**

Responses from 79 MND NSW members indicated that 15-21% had difficulty with speaking, writing and/or using a keyboard. Commonly used devices were desktop computers, laptops, tablets and mobile phones. Most participants (84%) were connected to the Internet and used it for email (91%), to find out more about MND (59%), to follow the news (50%) or for on-line shopping (46%). A third of respondents used Skype or its equivalent, but few used this to interact with health professionals.

**Conclusions:**

People with MND need greater awareness of technology options to access the most appropriate solutions. The timing for people with MND to make decisions about technology is critical. Health professionals need skills and knowledge about the application of technology to be able to work with people with MND to select the best communication technology options as early as possible after diagnosis. If people with MND are willing to trial telehealth technology, there is potential for tele-consultations via Skype or its equivalent, with health professionals. People with MND can benefit from health professional involvement to match technology to their functional limitations and personal preferences. However, health professionals need a comprehensive understanding of the application of available technology to achieve this.

## Introduction

The loss of the ability to communicate by speech, facial expression or hand gestures is one of the most devastating aspects of motor neuron disease (MND) [1]. Communication difficulties affect the majority of people with MND at some stage of the disease, and as such, assistive technology is essential for enabling them to continue with their everyday activities [2]. Likewise, telehealth and online communications are often important lifelines when leaving home or travel becomes too risky or difficult.

This article reports on an exploratory survey of people with MND about their use of communication technology, including generic and assistive technology. Assistive technology is defined broadly as any piece of equipment that is used to increase, maintain or improve function for people with disabilities [3], and will include communications technology. In the context of rapidly changing technology, public debate about telehealth, and projects such as the rollout of the National Broadband Network (NBN) in Australia, this project aimed to identify the current use of technology by people with MND, their attitudes towards technology use and how technology supports their communication needs.

### Literature Review

#### Motor Neuron Disease and the Needs of People With Disabilities When Identifying Technology Solutions

Motor neuron diseases (MNDs) are a group of progressive neurological disorders that destroy motor neurons, the cells that control essential voluntary muscle activity such as speaking, walking, breathing, and swallowing. In adults, the most common MND is amyotrophic lateral sclerosis (ALS), also known as Lou Gehrig’s disease. It is a degenerative condition characterized by damage to the motor neurons in the brain cortex, brainstem & spinal cord, and can involve both upper & lower motor neurons. Commons symptom include muscle wasting of the hand and feet muscles leading to foot drop, weakness and atrophy of the lower and upper limbs, fasciculation or involuntary muscle twitching, bulbar signs in the muscles of the palate, pharynx, & larynx leading to swallowing and speech problems. Generally, intellect, memory, sight, hearing, touch and taste remain intact, unless an individual is affected by fronto-temporal dementia. ALS affects adults and usually more men than women with an average age of onset of 58 years, usually when people are at highly active stages of their lives. Life expectancy is typically short (around 20-48 months) after diagnosis, combined with rapid loss of function, making the implementation of technology solutions very urgent [4,5].

Being able to create an optimal match of the needs of a person with a disability with technology solutions as early as possible, and involvement of the consumer in decision-making about the selection of the assistive technology solution are both essential for a successful outcome [6]. Such processes may help prevent the high levels of dissatisfaction with and non-use of technology solutions by people with disabilities [7]. This can be a very complex process, as the availability and development of potential technological solutions are constantly expanding, and reactions to physical and sensory changes associated with a disability have to be accommodated. Individual personalities, attitudes, past experiences, cultural values, environments, perceived capabilities and functional levels all have to be considered [8]. This is particularly true for technology to assist with communication, but people with MND are also likely to be faced with technology use in other areas of their lives, such as mobility, daily living tasks and home modifications. Therefore, the early use of technology has to be balanced by adjustments of people with a disability, as well as issues of grief, loss and identity.

#### Communication Needs and Solutions for People With MND

Due to the inconsistency of symptoms and the speed of deterioration in function, many people with MND are unprepared for the disabling loss of communication and the need to use assistive technology for communication [9]. The individual level of functional disability affecting communication and individual capabilities to use technology solutions are both likely to change throughout the progression of MND. This complicates potential intervention decisions and increases the learning demands for people with MND [10]. Augmentative and alternative communication (AAC) is defined as any mode of communication other than speech and includes low-tech as well as electronic communication devices [9]. Research on the attitudes and acceptance of the use of AAC and other technology in a range of communication settings is limited. However it is not uncommon for users to utilize more than one access strategy [11].

Literature indicating preferred communication hardware for people with MND is limited. Online forums for people with MND indicate a preference for lightweight, portable options, particularly the iPad or tablet computer. The most common difficulty of these devices is their inability to support adaptive equipment, so their useful life spans are short [12]. Current communication technology options include speech synthesis software for desktop, laptop and tablet computers, portable amplifiers, digital recorders, email and message boards [13]. Although there are several high-tech adaptive devices to use with computers such as SmartNav, eyegaze technology and the brain-computer interface [14], they all require extensive user training. The challenges with eye gaze interfaces are shared with other interfaces. For instance, the eye gaze technique is reported to be inaccurate in the selection of small objects, effortful and difficult to master, as well as being difficult to calibrate and expensive [15].

People with MND have reported that communication technology is essential to develop and maintain social closeness, and this is more important to them than the transfer of information to express needs and wants [11,16,17]. As a result, low-tech solutions may be adopted over high-tech equipment in many instances.

One common platform that can be used for social contact or to access health interventions is Skype (a voice over Internet protocol, or VoIP platform, with video capability). However, a review of research concluded there was no firm evidence in support of or against the use of Skype for telehealth [18]. Regardless of the platform chosen, the use of telehealth is expected to double in the next decade [19]. The advantages of VoIP include lower costs of providing care within the client’s own environment. The disadvantages include privacy, security and confidentiality risks [20,21], technological challenges and barriers to access such as cost, lack of access to Internet, low end-user technological literacy and confidence [22], and the preference of some clients for face to face consultations [23]. Telehealth has been used for assessment and rehabilitation in speech pathology, with clients reporting high satisfaction with the process [24]. Some consumers are also willing to adopt eHealth solutions despite some challenges in service dissemination [25].

Unfortunately, sometimes access to the appropriate information to engage with communication technology is particularly difficult for those who need it most [26]. Certainly the trend towards an “information society” brings the risk of a widening gap between those with access to technology and those without [26]. The Australian government NBN rollout is expected to extend the use of telehealth to aged, palliative and cancer care services as mainstream consultation options [27]. While the health system moves into the information age, it is assumed that consumers are keeping up with the pace.

Literature highlights the importance of early education and decision making about communication technology in recognition of the need and potential of various devices for people with MND [9,11]. Caregivers, family, doctors and allied health professionals are recognized as important contributors to this process, which should begin well before AAC is needed as a substantial communications support. Ultimately, consumer resistance may be the biggest challenge in achieving AAC solutions for people with MND. The use of a device for communication is perceived by some as “giving in” to the disease, and reflects a constant reminder of what the person has lost [9].

Therefore, this exploratory study aimed to investigate the types of technology (hardware and software) used by people with MND to communicate, their confidence and skill levels relating to technology, their perceived barriers to the use of technology for communication and their willingness to modify or update modes of communication, especially when interacting with support organizations and health professionals.

## Methods

A cross-sectional self-administered online survey was developed as a time and cost-efficient method of gathering data from people with MND who may have motor and speech difficulties. The survey was distributed to the Motor Neuron Disease Association of New South Wales (MND NSW) members. MND NSW is a non-government organization that supports people with MND throughout NSW, and is the peak body representing the interests of people with MND in New South Wales. Ethical clearance was obtained for the study from the University of Sydney Human Research Ethics Committee.

### Survey

The researchers completed a 26-module Web-based MND training course for professionals prior to developing the survey [28] to ensure they fully understood the key issues for people with MND. The 20-item technology survey encompassed three major themes: communication technology devices including AAC (eg, desktop and tablet computers), information sourcing (eg, Internet, social media) and communication methods (eg, email, VoIP). The objective was to collect detailed, specific data across a wide spectrum of topics without tiring the participants, so many questions had multiple tick box options. The draft survey was tested amongst the authors and piloted with informal contacts before being reviewed by MND NSW staff with expertise in the needs of people with MND. The final survey contained 18 closed-ended questions, each with space for free text comments, and 2 open-ended questions for free text responses at the conclusion of the survey. The survey can be seen in Multimedia Appendix 1. SurveyMonkey was chosen as the platform for the Web-based delivery of the survey system.

Study participants were given a choice of response methods depending on their preferences and capacity: (1) completing the survey online, independently, (2) completing and returning a mailed hard copy of the survey or (3) verbally responding to questions with a researcher by telephone. Questions were identical across all response methods.

### Procedure

MND NSW members who had responded positively to a “consent to contact” question in the annual MND NSW Member Satisfaction Survey (N=447), were invited to participate in the study by distribution of a participant information statement and consent form by MND NSW staff. Consenting participants indicated if they were willing to be contacted by researchers, and identified their preferred method of contact on a consent form. MND NSW staff distributed hard copies of the survey and reply-paid envelopes to participants requiring them, and sent an email to participants requesting the link to the Web-based survey. MND NSW staff provided researchers with the contact details of participants requesting a telephone interview to complete the survey. The survey remained open for 2 weeks.

All MND NSW members who were living with MND were eligible to participate. Carers were also eligible if they spoke on behalf of the person with MND.

### Data analysis

Survey data were downloaded in Excel, coded, and entered into SPSS. Descriptive statistics were used to summarize frequencies and cross tabulations. Free text data or participant responses from telephone interviews to the open ended questions were consensus coded and analyzed using thematic analysis [29].

## Results

Of the 93 members of MND NSW who consented to be contacted, 57 requested the online survey link, 27 requested a phone survey/interview and 9 requested a postal survey. A total of 79 completed surveys were returned. Of these, 70% (55/79) responded on line, 27% (21/79) responded by telephone interview and 4% (3/79) by mail. See Table 1 for further details.

**Table 1:** Responses to the survey.

Survey delivery method	Members agreeing to be contacted	Surveys completed
Mailed	9	3
Accessed online	57	55
Telephone	27	21
Total	93	79

The MND NSW membership was 447 at the time of the survey and 79 responses represented 20% of the total membership. As the survey was anonymous, we were unable to determine the characteristics of those members who did not participate in the study.

### Characteristics of Survey Respondents

Respondent age, gender and geographical distribution closely aligned to the overall MND NSW membership (see Table 2). However, there was an under-representation of those diagnosed within the previous 6 months (4.3% of respondents compared with 12.1% of the MND NSW membership) and an over-representation of those diagnosed for 3-5 years (21.7% of respondents compared with 14.5% of MND NSW membership).

### Use of Communication Technology

Most respondents (66/79, 84%) indicated that they used some form of aid or equipment for speaking and/or typing and/or handwriting, and Table 2 indicates the range of equipment used across these 3 communication modes. Of those surveyed, 4 respondents were unable to communicate in any mode (speaking, writing or typing), without assistance. Table 3 shows that more respondents aged 50-69 had impairments across the communication modes. Fewer respondents aged 70 and over were using any aids or equipment for communication.

### Technology and Devices Used

Most respondents (65/79, 82%) owned either a desktop or laptop computer, and 21% of the total group (16/79) owned both. There were no differences in usage between rural and urban respondents. The remaining 18% of respondents (14/79) did not have access to a computer in their home, and none indicated that they were borrowing either a desktop or laptop computer. Tablet computers were used by 33% (26/79) of respondents. Most tablet owners were female (17/26, 65%), and 5 respondents indicated they had a desktop computer but would prefer a laptop or iPad.

Webcams were the most popular assistive device, used by 10 respondents (see Table 2). Free text and verbal comments were provided by 57 respondents about devices used to augment speech. Of these, 9 respondents indicated they used speech Apps (such as SpeakIt, Verbally, Prolo2go and SayIt) and 2 used computer programs (NaturalSoft and E-triloquist). SpeakIt was the most frequent app identified by name by 5 respondents. These apps and programs were used on a range of devices. Laser head pointers and hands-free computer mice were used by 3 respondents, while 5 indicated they used boards or cards to assist with communication.

### Internet use

The majority of respondents (66/79, 84%) had access to the Internet at home, with 94% (74/79) having a broadband connection; 8 respondents did not have Internet access. Of these, 4 were aged over 70, 2 were 60-69 and 2 were 50-59, with half of them reporting they did not have the physical ability to use a desktop computer. One person commented that they used the National Relay Service via the Internet and 1 respondent commented their iPad use had changed since obtaining a PocketWifi, stating, “Fantastic. Can use my iPad when away from home. Previously only used iPad for Speakit application.”

Many respondents (n=54) indicated that they had used the Internet for email (49/54, 91%), to find out more about MND (32/54, 59%), news (27/54, 50%) and online shopping (25/54, 46%). Respondents reported an increase in time spent on the Internet since their MND diagnosis (23/54, 43%). Two respondents commented that the Internet was a way to fill in time as their physical ability became restricted by MND, making statements such as “It’s a pretty big part of filling my week now. I’d be pulling my hair out with boredom without it” and “Inactivity has meant more time for using the Internet”.

**Table 2:** Characteristics of survey respondents (N=79).

		Survey (N=79)		MND NSW Members (N=447)	
		n	%	n	%
**Gender (n=79)**					
	Male	41	52	257	57.5
	Female	38	48	190	42.5
**Location (n=68)** ^a^					
	Metropolitan	37	55	256	57.3
	Regional	8	12	70	15.7
	Rural	20	29	93	20.8
	Interstate	3	5	28	6.2
**Age (n=70)** ^a^					
	<40	2	3	17	3.8
	40-49	5	7	39	8.7
	50-59	18	26	77	17.3
	60-69	21	30	137	30.6
	≥70	24	34	177	39.6
**Length of MND diagnosis**					
	<6 months	3	4	55	12.2
	6-12 months	12	17	52	11.6
	1-3 years	23	33	152	34.2
	3-5 years	15	22	66	14.5
	>5 years	16	24	122	27.5
**Needing help or equipment with communication tasks**					
	Speaking (n=66)^a^	32	21		
	Handwriting (n=61)^a^	28	17		
	Typing/keyboard (n=58)^a^	27	16		
**Technology being used (n=61)** ^**a,b**^					
	Desktop or fixed computer	32	52		
	Laptop or notebook computer	33	54		
	Tablet (eg, iPad)	26	43		
	Mobile phone	49	80		
	TTY phone	2	3		
	Light writer	6	10		
	Message mate	1	2		
**Assistive technology being used (n=14)** ^**a,b**^					
	Webcam	10	71		
	Laser head pointer	3	21		
	Hands free computer mouse	3	21		
	Switch adaptation	2	14		
	Trackball computer mouse	1	7		
	Eye gaze	1	7		
	Specialized mounting	1	7		
**Sources of advice about technology (n=64)** ^**a,b**^					
	Family	44	69		
	Friends	29	45		
	Internet	27	42		
	MND Association	20	31		
	Speech therapist	18	28		
	Occupational therapist	9	14		
	GP	6	9		

^a^Some respondents did not answer all the survey items

^b^Respondents could select more than one response

**Table 3:** Age and selected survey responses (N=79).

		Under 40		40-49		50-59		60-69		70+	
		n	%	n	%	n	%	n	%	n	%
**Needing help for communication** ^a^											
	Speaking (n=32)	1	3	3	9	10	31	13	41	5	16
	Handwriting (n=28)	2	7	1	4	10	36	13	46	2	7
	Typing/ keyboard (n=27)	2	8	2	8	8	30	9	33	6	22
**Use of devices for communication** ^a^											
	Desktop computer (n=32)	1	3	3	9	10	31	9	28	9	28
	Laptop/Notebook (n=33)			2	6	14	42	10	30	7	21
	Tablet (eg, iPad) (n=26)			2	8	10	39	7	27	7	27
	Mobile phone (n=49)	1	2	4	8	14	29	15	31	15	31
	TTY phone (n=2)							1	50	1	50
	Light writer (n=6)					4	67	2	33		
	Message mate (n=1)							1	100		
**Already using email to contact others** ^a^											
	Neurologist (n=16)	1	6			6	38	8	50	1	6
	GP (n=8)					3	38	3	38	2	25
	Other medical specialist (n=5)					2	40	2	40	1	20
	Other health professional (eg, OT, ST) (n=12)			1	8	6	50	4	33	1	8
	MND Association (n=20)			2	10	7	35	8	40	3	15
	Other people with MND (n=12)			1	8	6	50	2	17	3	25
	Friends and family (n=39)			4	10	11	28	14	36	10	26

^a^Some respondents did not answer this section of the survey.

### Advice Sources and Support Requirements

Family members were the most common source of technology ideas and advice (44/64, 69% ) especially children (see Table 2), as illustrated in quotes such as “I ask my kids. Our age group is pretty illiterate about this stuff, they’re useless” and “I got the cleaning lady’s 14 year old son to help me out with the iPhone”.

Friends and the Internet were also popular sources of technology advice. MND NSW was selected by 31% (20/62) of respondents as a source of support.

Of allied health professionals, speech therapists were the most common sources of advice, followed by occupational therapists. One respondent commented that their occupational therapist was *“*really terrific with equipment but doesn’t address technology. I could use more support in this area”.

Comments from 5 respondents suggested a need for more general assistance with technology, but they were not clear who should provide this assistance, stating things such as “We are really in need of an in-depth consultation with someone who is really an expert in this area”.

Respondents indicated that they did not have sufficient expertise to know good technology choices to improve their function, and expressed frustration that there wasn’t a *“*one-stop shop*”* for ongoing assistance. One person had been unable to use the technology they had acquired; a caregiver stated that they “have been supplied with the Eyegaze but am yet to try it as we are unable to install it – we need someone to give him a demonstration”. An avoidance of seeking support or information, largely due to difficulty adapting and accepting a diagnosis of MND was expressed by 4 respondents, through such statements as “I think the thing is it is very early in my diagnosis so I have my head in the sand. I sort of hope they have made a mistake”, and “He’s aware of them (apps) but doesn’t want to adapt his lifestyle in any way, he doesn’t want to acknowledge the MND. He’s afraid if he does he’ll sort of go downhill”.

### Confidence and Skill Level Relating to Technology

**Figure 1:**
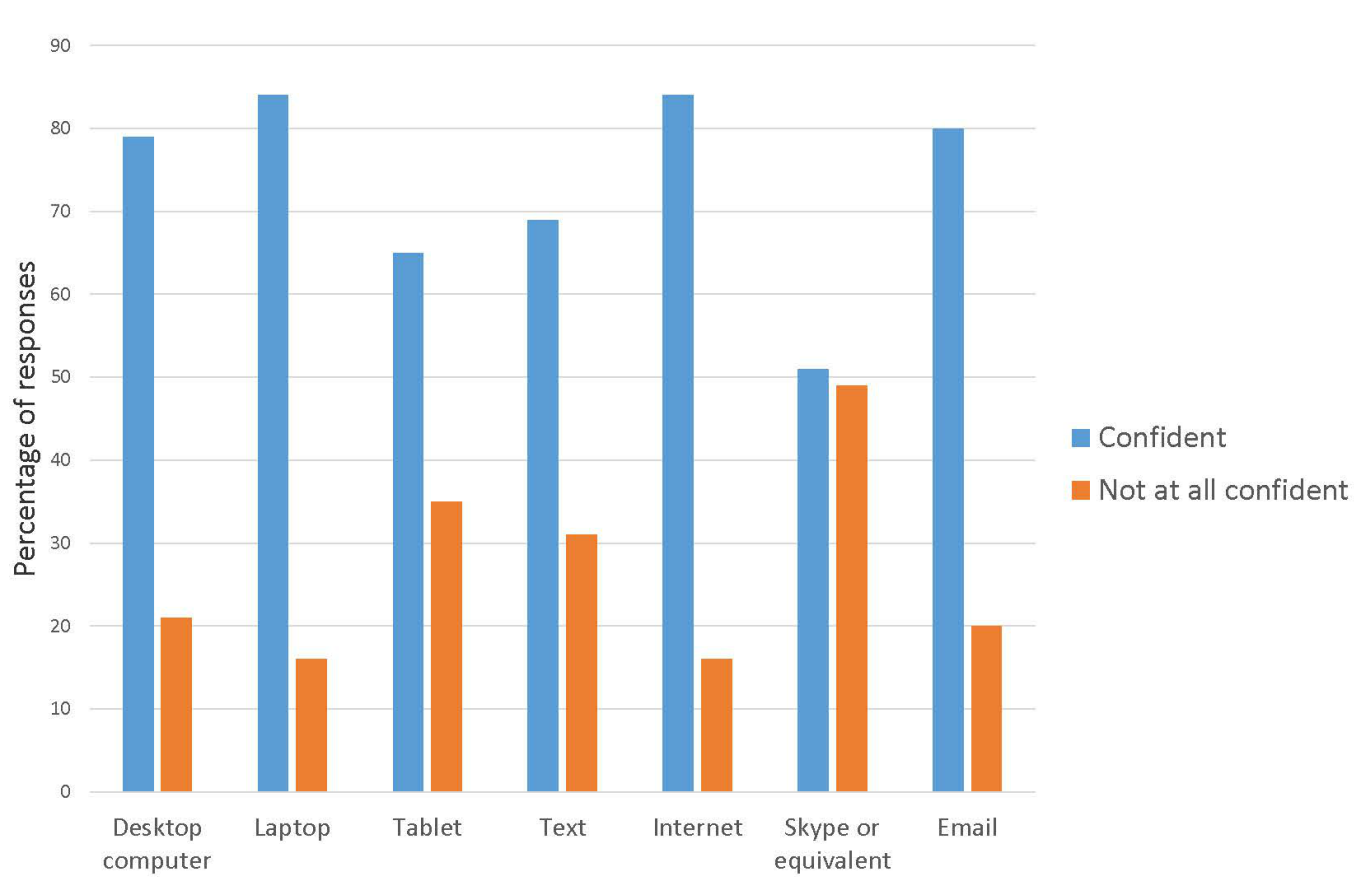
Ratings of confidence in using technology devices for communication (N=64).

Overall, respondents were considerably more confident than not with all forms of technology identified in the survey (Figure 1).

However, levels of confidence were related to age. Respondents aged 30-49 were confident in all forms of technology, although this age group had a low survey participation rate (n=5). Those aged 50-70 plus (n=63) were reasonably confident using desktop computers (47/63, 75%), laptop computers (52/63, 82%), the Internet (51/63, 80%) and email (49/69, 78%). However, they were less confident in using tablet computers (39/63, 62%) text messages (41/63, 65%) and video phones (32/63, 51%). Overall, 27% of people (8/30) identified a lack of confidence with technical skills as a reason for not using technology, however the response rate was low for this question (30/79, 38%).

When asked about adaptive devices, 2 respondents owned SmartNav or a laser head pointer but were unable to use them. One respondent had an Android tablet and an iPad, and found the Android version more difficult to use.

### Barriers to Use of Communications Technology

Only 30 respondents (30/79, 38%) answered survey items about barriers to communication technology use. Of those who responded, the most common barrier identified was a lack of physical ability (12/30, 40%), and 4 of these indicated they had limitations of hand or speech function. Others (5 participants) offered comments related to their capacity to use technology, such as “When she was able she used a button to press for attention and a laser head pointer to type - that was fabulous. She was able to do emails and banking when she had head control which she no longer has” and “The email and Internet was a fantastic source and outlet for me when I could operate it independently. Since my hands ceased being able to move I have been isolated from this and have to rely on my family to do any searches, research or emails”.

A high proportion of respondents (74/79, 93%) identified the need for support with technology, programs, hardware and/or adaptive equipment as a barrier to their use of technology. Cost was selected as a barrier by 7 respondents, regardless of the type of computer. One respondent identified the cost of apps as a prohibitive factor, stating “I do have them (apps) on my iPad, but rarely use them, the good ones are expensive to purchase, the ones I have are the free apps”. Lack of interest was identified as a barrier by 3 respondents, for instance, “He’s confident with the programs he knows but not interested in learning how to text or email”. A lack of computer literacy was mentioned by 3 respondents.

### Willingness to Adopt Use of Technology

Respondents were asked how willing they would be to use email and Internet video phone programs such as Skype to communicate with health professionals and others, if provided with the necessary equipment and skills. Overall, respondents were likely to consider using email (53/64, 83%) and video phone (53/64, 81%) with their friends and/or family, health professionals and MND NSW. Rates were lower for the potential of using these forms of communication with their GP, and 25% (16/64) indicated they would never email their GP, and 33% (21/64) would never use Skype to communicate with their GP.

When related to age, Table 3 indicates that email was already used by many respondents as a communication strategy. The lowest use of email was with GPs and medical specialists, and only 12 (12/64, 19%) of respondents used email to contact health professionals such as occupational therapists and speech therapists. However, most respondents indicated they were willing to consider the use of email to contact allied health professionals in the future.

**Figure 2:**
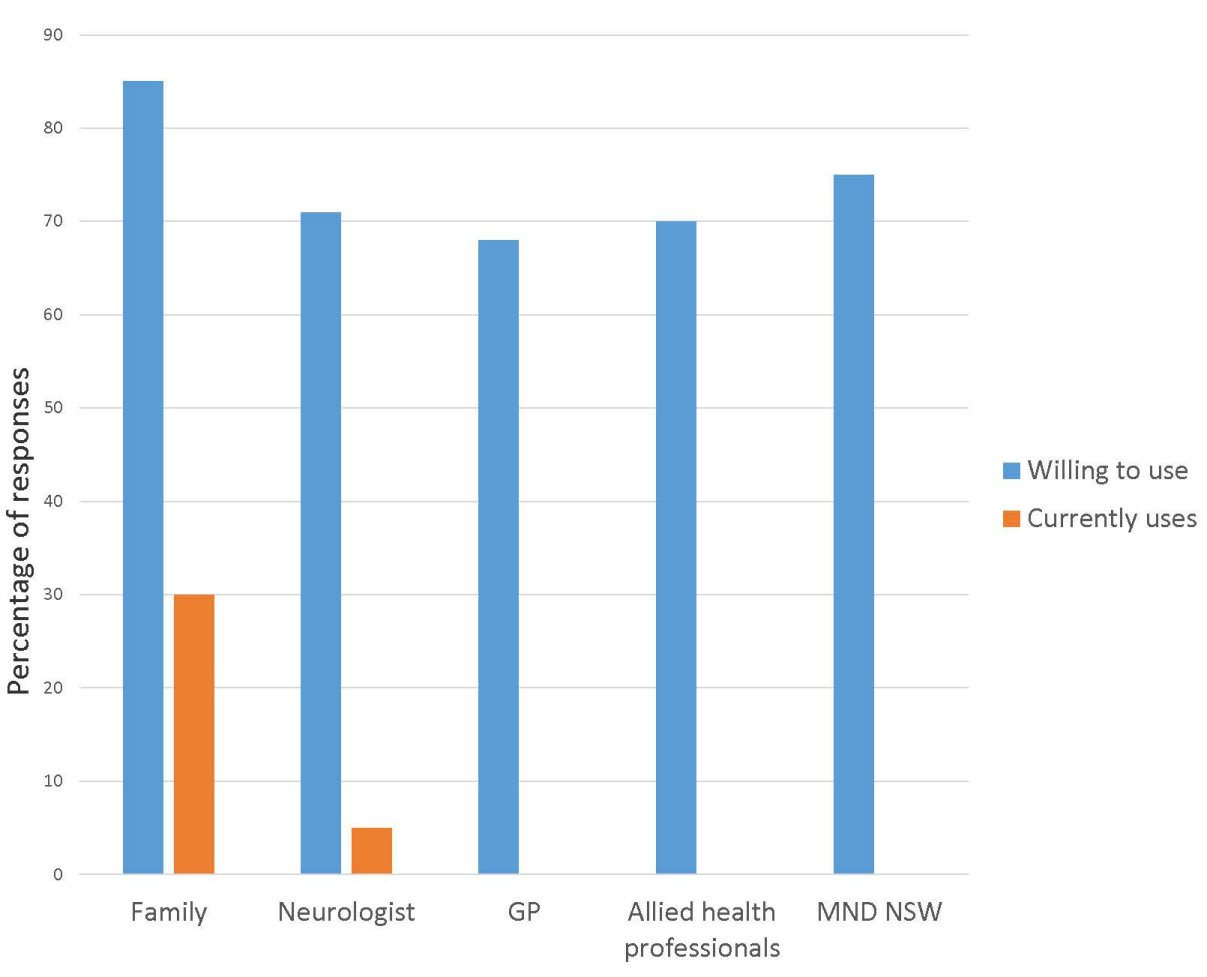
Ratings of willingness to use and usage of Skype or equivalent telehealth technology (N=64).

The use of Skype (or equivalent) had different results (see Figure 2). Only a third of respondents already used Skype with friends and/or family (19/64, 30%) and fewer with their neurologist (3/64, 5%). However no respondents indicated they used this technology to contact other health professionals or MND NSW. This was in contrast to members’ willingness to use Skype, which was much more positive overall (see Figure 2).

Comments were offered by 6 respondents who specified that they preferred face-to-face communication, and were reluctant to accept email or Skype as an alternative, making statements such as ”I have never done that and don’t think my computer is sophisticated enough to do that. I don’t think they would want me to do that… If they wanted me to, I guess maybe, but it would have to be them asking me”.

Data from the question relating to confidence were compared to that on willingness. While 12 respondents identified that they were not confident in using email, only 3 indicated that they would never email friends and family. This indicates that despite a lack of confidence, 9 people would be willing to email, suggesting a need for training to close this gap. Similar trends emerged for use of Skype or equivalent. While respondents indicated willingness to use this technology to communicate with a range of people (see Figure 2), only 50% (32/64) indicated they had a level of confidence in this technology (Figure 1). Just 10 respondents indicated that they would never be willing to use this to contact friends and/or family.

Tablet users (n=26) had higher email usage rates that users of other computers and while no-one in this group was using Skype or an equivalent technology with allied health professionals, 8% (2/26) were using it with their neurologist and 27% (7/26) were using it with friends and/or family.

### Preferences for Management of Communication Technology

Free text and verbal comments indicated that some respondents were unprepared for their speech loss when it occurred, and this was when the provision of equipment and training became urgent. Therefore, some expressed a preference for early technology interventions. For instance, one carer for a person with MND noted that “I’d be a great advocate for people to start learning technology immediately, because that’s the only type of communication you’re going to have. Communication is so hard with people with MND”.

When asked for additional comments relating to communications technology considerably more respondents discussed their frustrations (n=10) than the benefits (n=5). A need for additional support was expressed by 4 respondents, while 2 felt totally overwhelmed and 2 said they chose not to change their behavior because of their disease. However, 5 respondents expressed their gratitude for technology, and 8 respondents stated how technology was an important tool for facilitating communication and reducing isolation, making such statements as “The biggest thing for me is that MND stops a person being independent, but with a computer (she) was able to communicate, interact and be very much a part of our life – so grateful that she had this equipment”, ”Without email, typed notes and text messages I would be unable to communicate my thoughts, wishes, and I would be unable to take care of my own affairs”, and “To be disabled without technology is unthinkable”.

## Discussion

The study met the objectives of exploring the use of communication technology by people with MND, their confidence relating to technology, their perceived barriers to the use of technology for communication and the willingness of people with MND to adopt modes of communication or participate in telehealth. Study results have highlighted a number of opportunities for service providers and support organizations to direct their efforts in promoting communications technology for this client group.

### Awareness of Options for Communication Technology

Study participants identified physical limitations as a barrier to technology use, although they tended not to anticipate needing assistance in this area. This suggests a need for greater awareness of the benefits of technology early on following diagnosis, as well as knowledge of the types of adaptive devices available. This will enable people with MND to access appropriate technology in a timely manner. This is consistent with other studies suggesting that health professionals need to prepare people with MND to recognize the need and potential of communication technology, and these discussions should begin as early as possible after diagnosis [9,10]. However, involving people with MND in the decision-making about technology use early on can be a complex issue for a population of people where skills can rapidly decline. Raising awareness and providing training so that technology can be taken up may be a solution, but the process has to fully account for the psychosocial adjustment of people with MND to inevitable feelings of loss and grief as their personal identity is under threat [6,7]. Carers and health professionals should collaborate in preparing people with MND for this technology [10], and carer involvement is critical, as evidenced by the data from this study.

Any awareness-raising activities should be ongoing as individual needs change, and should take into account unpredictable progression rates and different forms of MND [10], as well as any resistance to acquiring technology. It has been suggested that most people with MND reluctantly accept the need for medical equipment to manage MND, however communications technology is seen as “giving in” [11]. This sentiment was echoed by the survey respondents. Many people with MND and their families may develop their own successful, no-tech solutions for dealing with communication loss [17]. Despite difficulties with distance or mobility and potential solutions using communication technology, many individuals will always prefer face-to-face communication [23].

### Use of Skype or Equivalents and Telehealth Technology

The willingness of respondents to use video Internet technology to communicate with family and/or friends suggests that this technology could also be used to ensure levels of social communication and support for people with MND, and to address any isolation associated with MND. Greater social use of this technology may or may not lead to greater acceptance of technology for communicating with professionals. However, such technology will support the primary objective of communication to develop and maintain social relationships [17].

The findings also highlighted the willingness of people with MND to trial telehealth technology options. High rates of broadband Internet connection suggest that Internet connectivity is not a barrier to telehealth. Some health professionals use tele-consultations via Skype with MND clients as a practical form of communication for this client group. Palliative care is another clinical area that is currently a priority for national telehealth trials and growing opportunities for remote consultations [27]. However, study findings support an initial face-to-face visit prior to implementing this technology.

Implementation of telehealth technology would require training for health professionals both around the use of the technology and the ethical issues surrounding remote consultations. A randomized controlled trial demonstrated that remote consultations were less acceptable to patients than face to face visits, and security issues remain a barrier to expansion of telehealth [20,21,23]. Despite access to the Internet and a willingness to try video Internet technologies, findings indicated that people with MND lack the confidence and/or skills to utilize these technologies, so further training and support is needed.

### Communication strategies

Study participants already interacted with technology to some extent, as the majority of surveys were completed online, and participants indicated the use of many communication technologies. This suggests that the use of Web-based communication for people with MND should be developed further, and the Internet provides opportunities to deliver education and support. Streaming sessions such as webinars with MND experts could offset any inaccurate online information related to MND [30]. Online support groups could be a worthwhile strategy for people with MND and their carers who, due to personal preference and/or the effects of MND, are unable to attend a face-to-face support group. While such technology cannot fully replicate the support of face-to-face meetings, it may be a valuable tool to supplement meetings and ensure inclusion of remote or isolated people with MND.

Findings also demonstrated that people with MND need more information about communication technology provided by reputable sources, rather than searching online. As respondents indicated that they did a lot of online searching for information around the time of their diagnosis, access to accurate and helpful information at this stage is important.

### The Role of Health Professionals

Study participants were less likely to access professional support for technology than asking their family and friends. However, the variety of devices and apps available suggests a need for some professional support in selecting the most suitable technology solution to fit individual circumstances. The challenge for health professionals is ensuring that awareness, referral and interventions are appropriately timed so that technology adoption is more likely [11]. Findings suggest that people with MND are using a range of communication options from very basic to high-tech solutions. Health professionals need to recommend communication strategies that require a minimal challenge in terms of new learning as the disease progresses [11]. Regular review and monitoring should be prioritized to ensure technology that is no longer useful is replaced with appropriate alternatives.

Occupational therapists and speech therapists are considered to be central to the process of assessing for and recommending technology solutions for people with MND (and others), in particular examining access to technology and capacity to operate it (movement, reach, endurance, hand function etc), seating, and visual and cognitive issues [5]. In order to fulfill such roles, therapists need to be knowledgeable about the variety of technology solutions available, both in mainstream technology as well as more specialized applications, and how they can be adapted for use by people with a variety of functional and progressive limitations [30). However, there is evidence that knowledge and skills in technology applications are not well developed [31]. The World Health Organization reported that health professionals were not sufficiently skilled to manage the needs of people with chronic conditions such as MND, with one defined skills being the ability to implement information and communication technology [32].While general skills in use of technology may have developed over the last 10 years, and new health professional graduates have skills in social networking and mobile phone use, this may not transfer to competence in implementing technological solutions for people with MND [33].

Provision of electronic assistive technology is regarded as a specialist area due to the sophistication of some technological solutions. Successful provision also requires the capacity to navigate complex local systems of funding [31]. Surveys of occupational therapists in Ireland and the UK indicate that while technology is viewed as an important component of their role, many are not confident about their competence to implement solutions, and identified training needs both at a preparation course level and at a continuing professional education level [31,34]. Little information is available on the competence and practice of Australian health professionals in providing technological interventions.

### Limitations of the Study

This study used cross-sectional survey methods and therefore could only provide information on responses to structured questions at one point in time. Not all survey items were mandatory, which allowed a low response rate for some questions. Although free-text comments were encouraged throughout the survey, use of individual interviews may have provided more in-depth information. The response rate to the survey was low, and it is unclear if the 80% (358/447) of the potential respondent pool from the MND NSW membership who did not participate were systematically different to the study sample in their use of technology. It is also possible that as MND NSW membership is voluntary, this organization may not include all people with MND in NSW. Furthermore, as 70% (55/79) of respondents selected the Web-based survey, this may bias results to those who are already using technology. Therefore, the results are not generalizable to the whole population of people with MND.

Bearing in mind the nature of the sample group, a low participation rate could be expected, as some potential participants may not have been able to tolerate the effort required to respond to the survey. This also raises the possibility of some systematic differences in the depth of data drawn from telephone interviews or non-representative views from carers responding on behalf of people with MND. However, taking into the account the limitations of surveying this population and giving all consenting participants the opportunity to have their views included by whatever ethical means were appropriate for them, we can be confident that the findings do represent the views of the sample.

### Conclusion and Recommendations

The survey findings indicated there were groups of respondents with different needs and preferences for communication technology. Some were early adopters of technology, with the skills, equipment and confidence to engage with technology. Others were willing but lacked either the confidence or skills to use technology, while some had access to equipment but were not willing to engage with the technology. For this sample, there appears to be a need for ongoing training and support in the use of technology to overcome a lack of confidence or skills in using the devices and software, and to maintain individuals as their disease progresses. To achieve this would require resourcing of technical support and expert advice. Ultimately, some people with MND will choose not to utilize communication technology, but there is an opportunity to target those who are willing to use technology but currently lack the necessary access to devices or skills to use them. All study participants would appear to benefit from the involvement of knowledgeable health professionals to create the right ongoing match between technology solutions, functional limitations and personal preferences.

The study results have identified recommendations for service providers to consider when addressing the needs of people with MND:

- Development of awareness-raising activities to allow opportunities for people with MND to adopt technology at an early stage.
- Training in technology devices for people with MND, particularly in the early stages of the disease, and targeted to those who are willing to use technology but currently lack skills or confidence.
- Provision of information on high-quality technology options to counter less helpful information derived from a free Internet search.
- A formal assessment by a qualified health professional is an important step in accessing effective solutions.
- Opportunities for health professionals to maintain and enhance their technology knowledge, so they can offer the best technology solutions matched to the needs of people with MND.

Future developments in technology are inevitable and will continue to challenge health professionals in working with people with MND with communication needs.

## Multimedia Appendix 1

MND Technology Survey.

## Abbreviations

AAC: augmentative and alternative communication
ALS: Amyotrophic Lateral Sclerosis
MND: motor neuron disease
MND NSW: Motor Neuron Disease Association of New South Wales
NBN: National Broadband Network
VoIP: voice over Internet protocol
