# Supplementary material for: Communications Technology and Motor Neuron Disease: An Australian Survey of People With Motor Neuron Disease
Source: JMIR Rehabil Assist Technol. 2016 Jan 25;3(1):e2. doi: 10.2196/rehab.4017 (PMC5454550; doi:10.2196/rehab.4017)
Supplement: Supplementary file 1 [file rehab_v3i1e2_app1.pdf]

Thank you for participating in this survey. By understanding which devices, networks and systems you are currently using MND NSW can continue to provide you with the highest quality of support now and in the future.These questions have been compiled by Master of Occupational Therapy students from Sydney University who are assisting MND NSW to find out more about members' use of communications technology.

If you wish, please write comments in the blank spaces provided. Your comments will help to clarify your answers and give us a clearer picture of your experiences. Estimated time for survey completion is 20 minutes. Your response is invaluable and we sincerely thank you for your time. All answers will remain anonymous.

1. Are you male or female?

- ◯ Male
- ◯ Female

2. What is your post code?

Post Code: ____________

3. Which category includes your age?

- ◯ 39 or younger
- ◯ 40-49
- ◯ 50-59
- ◯ 60-69
- ◯ 70 or older

4. How long have you been diagnosed with MND?

- ◯ Under 6 months
- ◯ 6 months to less than 1 year
- ◯ 1 to less than 3 years
- ◯ 3 to less than 5 years
- ◯ Over 5 years

5. Motor neurone disease can change the way a person communicates by affecting speech, handwriting, facial expression and hand gestures. Please indicate how, if at all, MND has affected your ability to communicate:

	I don't need help with this and I don't use aids	I don't need help with this but I use aids and/or equipment	I sometimes need help with this	I am unable to do this or always need help
Speaking	◯	◯	◯	◯
Handwriting	◯	◯	◯	◯
Typing/Keyboard	◯	◯	◯	◯

Please explain further the aids, modification or equipment you use and/or assistance you require:

6. People with MND use a range of devices to assist them with communication. Please select all the devices that you use and indicate how you acquired them.

	I bought it	I am borrowing it	It was a gift
Desktop or fixed computer	◯	◯	◯
Laptop/notebook computer	◯	◯	◯
Tablet computer eg iPad	◯	◯	◯
Mobile phone	◯	◯	◯
TTY phone	◯	◯	◯
Lightwriter	◯	◯	◯
Message mate	◯	◯	◯
Other	◯	◯	◯

Other, please specify:

7. Do you use any of the following to assist you? Please select all that apply:

- ☐ Webcam
- ☐ Laser head pointer
- ☐ Hands free computer mouse
- ☐ Eye gaze
- ☐ Switch adaptation
- ☐ Trackball computer mouse
- ☐ Specialised mounting

Other, please specify:

8. An increasing number of speech generating computer programs and communication apps are available to assist with communication eg: Proloquo2Go and Speak it. Do you use any communication computer programs or communication apps?

- ◯ Yes, please list the communication apps or programs used:
- ◯ No, please comment on why you are not using communication apps or programs eg: you are unaware of them, or not interested in using them:

Comments:

9. How confident are you with using the following:

	Not confident at all	Reasonably confident	Confident	Very confident
Desktop/fixed computer	◯	◯	◯	◯
Laptop/notebook computer	◯	◯	◯	◯
Tablet computer eg iPad	◯	◯	◯	◯
Text messages	◯	◯	◯	◯
Internet	◯	◯	◯	◯
Email	◯	◯	◯	◯
Internet video phone (VoIP) eg: Skype, FaceTime	◯	◯	◯	◯

Other (please specify)

10. Do you have access to the internet at home?

- ◯ Yes
- ◯ No

Other (please specify)

11. What type of internet access do you have?

- ◯ Broadband connection (including ADSL, Cable, Wireless and Satellite connections)
- ◯ Dial Up connection (including analog modem and ISDN connections)
- ◯ Other (include internet access through mobile phones, etc) please specify

Comments:

12. Has your internet use changed since your diagnosis with MND?

- ◯ Yes, it has increased
- ◯ Yes, it has decreased
- ◯ I do not use the internet
- ◯ No, it has remained the same

Comments:

13. On average, how much time do you spend on the internet each day?

- ◯ Less than 1 hour
- ◯ 1- 2 hours
- ◯ 3-4 hours
- ◯ More than 4 hours

Comments:

14. People use the internet for a range of purposes. We are interested in knowing what people with MND use the internet for. Please select all that apply:

- [ ] Accessing the National Relay Service
- [ ] Chat forum/discussion boards
- [ ] Email
- [ ] Finding information about MND
- [ ] Games multi user
- [ ] Games single user
- [ ] News
- [ ] Shopping
- [ ] Social networking eg Facebook
- [ ] Video Phone eg Skype or FaceTime
- [ ] Work
- [ ] Other eg entertainment, please specify

Comments:

15. Can you identify anything that prevents you from using a computer, laptop or tablet computer? Please select all that apply:

	Desktop/Fixed computer	Laptop/notebook computer	Tablet computer eg: ipad
I have one but don't use it	☐	☐	☐
It's too expensive	☐	☐	☐
I don't feel confident with my technical skills	☐	☐	☐
It is difficult to access the internet where I live	☐	☐	☐
I don't know what type of device would be suitable for me	☐	☐	☐
I don't know what type of programs/apps would assist me	☐	☐	☐
I don't have the physical ability to use it	☐	☐	☐
I'm not interested	☐	☐	☐

Other reasons, please specify:

16. Who or where do you get technology ideas and advice from? Please select all that apply:

- ☐ Family members
- ☐ Friends
- ☐ Computer shop/business
- ☐ MND Association
- ☐ Speech therapist
- ☐ Occupational therapist
- ☐ Physiotherapist
- ☐ GP
- ☐ Internet
- ☐ I don't need any advice
- ☐ I don't know where to get advice from
- ☐ Other, please specify

Comments:

17. If you had the necessary equipment and skills how willing would you be to communicate with the following people via EMAIL? Please select all that apply:

	Never	Maybe	Quite likely	Very likely	Already do
Neurologist	◯	◯	◯	◯	◯
GP	◯	◯	◯	◯	◯
Other medical specialists eg: Respiratory physician	◯	◯	◯	◯	◯
Other health professionals eg: Occupational therapist, Speech therapist, Nurse	◯	◯	◯	◯	◯
MND Association	◯	◯	◯	◯	◯
Other people with MND	◯	◯	◯	◯	◯
Friends/Family	◯	◯	◯	◯	◯

Comments:

18. If you had the necessary equipment and skills, how willing would you be to communicate with the following people via INTERNET VIDEO PHONE eg: Skype? Please select all that apply:

	Never	Maybe	Quite likely	Very likely	Already do
Neurologist	◯	◯	◯	◯	◯
GP	◯	◯	◯	◯	◯
Other medical specialist eg: Respiratory physician	◯	◯	◯	◯	◯
Other health professionals eg: Occupational therapist, Speech therapist, Nurse	◯	◯	◯	◯	◯
MND Association	◯	◯	◯	◯	◯
Other people with MND	◯	◯	◯	◯	◯
Friends/Family	◯	◯	◯	◯	◯

Comments:

19. Do you have any other comments on your use of communication devices and the internet since your MND diagnosis (for example unexpected benefits or frustrations)?

20. Do you have any other comments?

Thank you for contributing to our research. Your time and effort are very much appreciated.
